# High expression of Sterol-O-Acyl transferase 1 (SOAT1), an enzyme involved in cholesterol metabolism, is associated with earlier biochemical recurrence in high risk prostate cancer

**DOI:** 10.1038/s41391-021-00431-3

**Published:** 2021-07-29

**Authors:** Carolin Eckhardt, Iuliu Sbiera, Markus Krebs, Silviu Sbiera, Martin Spahn, Burkhard Kneitz, Steven Joniau, Martin Fassnacht, Hubert Kübler, Isabel Weigand, Matthias Kroiss

**Affiliations:** 1 University Hospital Würzburg, Department of Internal Medicine I, Division of Endocrinology and Diabetology, Würzburg, Germany; 2 University Hospital Würzburg, Department of Urology and Pediatric Urology, Würzburg, Germany; 3 University of Würzburg, Comprehensive Cancer Center Mainfranken, Würzburg, Germany; 4 Lindenhofspital, Bern, Switzerland; 5 Department of Urology, University Hospital Essen, Essen, Germany; 6 Department of Urology, University Hospitals Leuven, Leuven, Belgium; 7 Department of Medicine IV, University Hospital Munich, Ludwig-Maximilians-Universität München, München, Germany

**Keywords:** Prostate cancer, Prognostic markers

## Abstract

**Background:**

Prostate cancer (PCa) is the most frequent cancer in men. The prognosis of PCa is heterogeneous with many clinically indolent tumors and rare highly aggressive cases. Reliable tissue markers of prognosis are lacking. Active cholesteryl ester synthesis has been associated with prostate cancer aggressiveness. Sterol-O-Acyl transferases (SOAT) 1 and 2 catalyze cholesterol esterification in humans.

**Objective:**

To investigate the value of SOAT1 and SOAT2 tissue expression as prognostic markers in high risk PCa.

**Patients and methods:**

Formalin-fixed paraffin-embedded tissue samples from 305 high risk PCa cases treated with radical prostatectomy were analyzed for SOAT1 and SOAT2 protein expression by semi-quantitative immunohistochemistry. The Kaplan–Meier method and Cox proportional hazards modeling were used to compare outcome.

**Main outcome measure:**

Biochemical recurrence (BCR) free survival.

**Results:**

SOAT1 expression was high in 73 (25%) and low in 219 (75%; not evaluable: 13) tumors. SOAT2 was highly expressed in 40 (14%) and at low levels in 249 (86%) samples (not evaluable: 16). By Kaplan–Meier analysis, we found significantly shorter median BCR free survival of 93 months (95% confidence interval 23.6–123.1) in patients with high SOAT1 vs. 134 months (112.6–220.2, Log-rank *p* < 0.001) with low SOAT1. SOAT2 expression was not significantly associated with BCR. After adjustment for age, preoperative PSA, tumor stage, Gleason score, resection status, lymph node involvement and year of surgery, high SOAT1 but not SOAT2 expression was associated with shorter BCR free survival with a hazard ratio of 2.40 (95% CI 1.57–3.68, *p* < 0.001). Time to clinical recurrence and overall survival were not significantly associated with SOAT1 and SOAT2 expression

**Conclusions:**

SOAT1 expression is strongly associated with BCR free survival alone and after multivariable adjustment in high risk PCa. SOAT1 may serve as a histologic marker of prognosis and holds promise as a future treatment target.

## Introduction

Prostate cancer (PCa) is the most frequent cancer in men [1]. Apart from its high incidence, PCa heterogeneity poses a major challenge in clinical care. While the incidence of PCa has increased in the western world as a result of widespread screening programs using prostate specific antigen (PSA) as a serum marker [2], mortality rates did not decrease substantially [3]. This reflects that the majority of PCa patients has indolent (low risk) cancers while the small subgroup of lethal (high risk) cancers cannot be reliably identified and treated after positive PSA screening. Prognostic markers are hence of major relevance. The current strategy to identify high risk prostate cancer (high risk PCa) cases uses a combination of clinical and histological markers because it has been shown that PCa-related death can be predicted by using tumor stage 3b-4, Gleason score ≥8 and a positive lymph node status as markers of unfavorable prognosis [4].

Accumulating data suggest that features of tumoral metabolism may be prognostically relevant and be of therapeutic relevance [5]. By using RAMAN-spectroscopy of high risk PCa samples, accumulation of cholesteryl esters (CE) has been shown to be a hallmark of PCa progression. Experimental data suggest that loss of the tumor suppressor PTEN, a known driver of PCa, might be causally related to increased cholesterol metabolism [6]. Inhibition of cholesteryl ester formation with avasimibe, an inhibitor of Sterol-O-Acyl transferase 1 (SOAT1, also known as Acyl-CoA-Cholesterol Acyl transferase 1, ACAT1) in a cell culture model of PCa has been shown to reduce cell viability and in vitro indicators of cell migration and invasiveness. Subsequent research in a mouse model of metastatic PCa treated with injectable avasimibe showed that depletion of CE resulted in abrogated Wnt signaling [7] through an indirect mechanism involving suppression of fatty acid synthesis and decreased Wnt3 acylation. While the relationship of cholesterol metabolism and tumoral androgen synthesis in PCa is still controversial [8, 9], available data suggest that targeting CE formation may be a promising approach for treatment of aggressive PCa [6, 7].

Intracellular synthesis of CE in humans from cholesterol and Acyl-CoA is catalyzed by SOAT1 and SOAT2. According to current understanding, SOAT1 and 2 exhibit functional differences: SOAT1 is expressed at variable levels in many tissues and preferentially catalyzes the esterification of free cholesterol with unsaturated fatty acids [10] that are stored as intracellular lipid droplets (LD). LDs hence serve as buffers for free cholesterol, which would otherwise impair cellular function by interfering with membrane fluidity [11]. SOAT2 is mainly expressed in liver and intestine and plays a role in secretion of lipoproteins [12, 13].

We have previously shown that SOAT1 inhibition is the main molecular mechanism of mitotane, the only drug approved for treatment of adrenocortical carcinoma [14]. The selective SOAT1 inhibitor nevanimibe (ATR-101, PD132301-2) has been investigated for the treatment of adrenal disorders [15, 16] and was recently tested in a phase I clinical trial against adrenocortical carcinoma [17].

Protein expression of SOAT1 and SOAT2 has not been examined in prostate cancer tissue. In the present study, we investigated the expression of both enzymes in a large cohort of well characterized high risk PCa tissue samples from two centers and demonstrate strong association of SOAT1 expression with poor prognosis. We used time to biochemical recurrence (BCR), a strong predictor of cancer-specific mortality in high risk PCa, as the primary end point [18].

## Patients and methods

### Patients and tissue

305 formalin-fixed-paraffin-embedded (FFPE) high risk PCa samples (defined as PSA > 20 ng/mL and/or clinical stage T3/4 and/or biopsy Gleason score 8–10) who had undergone radical prostatectomy between 1987 and 2005 at two clinical centers (Karlsruhe, Germany: 206 samples, Leuven, Belgium: 99 samples) were included in this study. Detailed clinical data were collected in the European Clinical and Translational High-Risk Prostate Cancer Research Group database (EMPaCT) as described before [19]. All patients underwent preoperative staging with digital rectal examination (DRE), abdominopelvic computed tomography scan and bone scan. PCa was confirmed by transrectal biopsy and pretreatment PSA was measured before DRE. None of the patients had received neo-adjuvant radiation or chemotherapy. After surgery, the absence or presence of local recurrence or distant metastasis (clinical progression) was evaluated every three months for the first two years, every six months in the following three years and annually after by computerized tomography or bone-scan. BCR was defined by prostate-specific antigen (PSA) of greater than 0.2 ng/ml in two consecutive measurements. Overall survival (OS) was defined as time from radical prostatectomy to death of any cause or last follow-up visit, while cancer-specific survival (CSS) was defined as death caused by PCa or complications of PCa. With a known overall BCR rate of ~35% in our available high risk PCa cohort, 116 events would be necessary to observe a clinically relevant difference in the incidence of 25% vs. 50% BCR with a power of 80% and an alpha error of 0.05.

Written informed consent was obtained from all patients and the study was approved by the cantonal ethics committee in Bern (128/2015; 26.05.2015) and performed in accordance with the declaration of Helsinki. Median follow-up was 89 months (range 6–200).

Bioinformatical analysis of the TCGA (The Cancer Genome Atlas) Research Network, specifically its primary PCa (PRAD) cohort, and the DreamTeam cohort [20] consisting of PCa metastases was carried out with data retrieved from the cBioPortal [21].

### Immunohistochemistry

Tissue samples were assembled into five tissue microarrays (TMA) and up to four cores were available per sample and punch diameter was 0.6 mm. Slides of 4 µm were deparaffinized twice in 100% xylene (Sigma-Aldrich, Taufkirchen, Germany) for 12 min and rehydrated in a descending ethanol-series, followed by washing with distilled water. Antigen retrieval was performed in 10 mM citric acid monohydrate buffer (pH 6.5) for 13 min in a pressure cooker. Quenching of endogenous peroxidase activity was done with 3% H_2_O_2_ for 10 min. Unspecific binding sites were blocked with 20% human AB serum for 1 h at room temperature. Subsequently, SOAT enzymes were detected by incubation with polyclonal rabbit primary antibodies (SOAT1-antibody, abcam, Cambridge, UK, ab39327, 1:1000 in PBS over night at 4 °C; SOAT2-antibody, Acris Antibodies GmbH, Herford, Germany, Cayman Chemicals 100027, 1:400 in PBS for 1 h at room temperature). Signal amplification was achieved by using Advance-HRP-Kit (Dako, Hamburg, Germany) and development for 10 min with Liquid DAB + Substrate Chromogen System (K3468, Dako) according to the manufacturer´s instructions. Samples were counterstained with Mayer´s hemalaun for two minutes followed by washing in tap water.

### Microscopy and quantification of expression

All slides were analyzed independently by two investigators (C.E. and I.S.) by using semi-quantitative H-scoring as described [22]. Discrepancies in scoring were double checked by both investigators and a consensus reached.

Only cytoplasmic staining of tumor cells was considered and staining intensity graded as negative (0), low (1), medium (2), and strong (3). A proportion score was calculated by determining the percentage of positive tumor cells for each sample which was 0 if 0% tumor cells were positive, 0.1 if 1–9% were positive, 0.5 if 10–49% were positive and 1 if ≥50% were positive. H-score was then calculated by multiplying the staining intensity grading score with the proportion score (range 0–3) taking into account all TMA cores. H-score of <3 vs. 3 was considered as low and high SOAT expression, respectively.

### Statistics and bioinformatical analyses

The interobserver agreement was evaluated using Cohen’s κ. The Correlation between SOAT H-Score and histological Gleason score was analyzed using Spearman’s rank correlation test. Survival was estimated using the Kaplan-Meier method and groups were compared by log rank test. Cox proportional hazards modeling included age, tumor stage, preoperative PSA, Gleason score, resection status, lymph node status and year of surgery. The significance level was set as *p* < 0.05 for all comparisons. All statistical tests were performed using SPSS 26.0 (IBM, Armonk, NY) or Prism 6 (GraphPad, San Diego, CA). Co-expression analyses of SOAT1 and genes related to androgen signaling [23]. and cholesterol biosynthesis [24] were performed via cbioportal [21, 25]—Spearman´s rank correlation coefficients were considered significant after performing Benjamini-Hochberg correction.

## Results

### Patient characteristics

The study cohort is described in Tables 1 and S1. Median age at surgery was 66 years (range 41–81) and median histopathological Gleason score 7 (range 3–9). After prostatectomy, resection was considered complete (R0) in 123 patients (40.3%), R1 in 147 and unknown (RX) in 35 patients (11.5%). Median follow-up was 89 months (range 6–200) after radical prostatectomy. 109 patients experienced biochemical recurrence after a median of 26 months (range 1–166), 44 had clinical progression after a median of 36 months (range 3-144) and 22 died of PCa (57 months, 14–166).

**Table 1:** Clinico-pathological features of the study population.

Variable	Median/number
**Age at surgery** (range)	66 (41–81)
**Pre-operative PSA, ng/ml** (range)	30 (1–597)
**Clinical stage**	
T2	59 (19.3%)
T3	203 (66.6%)
T4	43 (14.1%)
**Gleason-score**	
≤7	222 (77.6%)
8-10	64 (22.4%)
**Resection margin**	
R0	123 (40.3%)
R1	147 (48.2%)
RX	35 (11.5%)
**Lymph node status**	
N0	211 (69.4%)
N1	93 (30.6%)
**SOAT1**	
**expression score**	
<3	219 (75.0%)
3	73 (25.0%)
**SOAT2**	
**expression score**	
<3	249 (86.2%)
3	40 (13.8%)
**BCR**	
In total	109 (35.7%)
**Median time to BCR**, month (range)	25.5 (1-166)
**Clinical progression**	
In total	44 (14.4%)
**Median time to clinical progression**, month (range)	35.5 (3-144)
**Death for any reason**	30 (10.0%)
**Cancer related death**	22 (7.2%)

*n* = 305; only data included with complete clinical data and evaluable tissu.

*PSA* prostate-specific antigen, *SOAT* Sterol-O-Acyl transferase, *BCR* biochemical recurrence.

### Immunohistochemical detection of SOAT

**Fig. 1:**
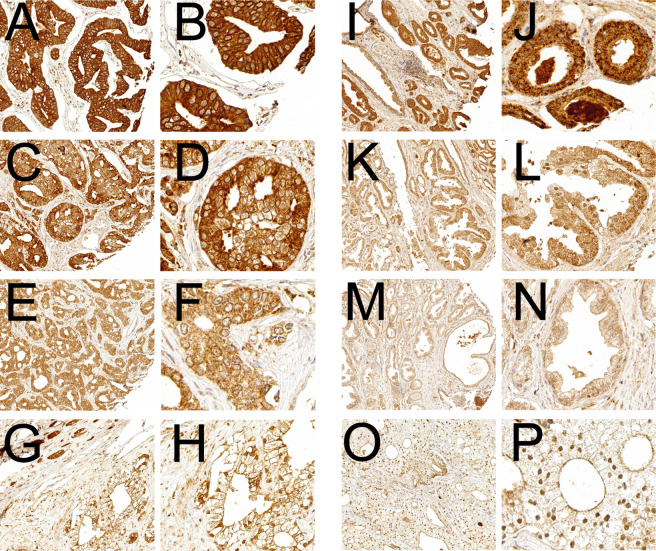
SOAT1- and SOAT2-immunohistochemistry of high-risk prostate-cancer. High-risk prostate cancer tissue with strong (H-score 3, A + B, I + J), moderate (H-score 2, C + D, K + L), low (H-score 1, E + F, M + N) and negative (H-score 0, G + H, O + P) expression of SOAT1 (**A**–**H**) and SOAT2 (**I**–**P**).

Both SOAT1 and SOAT2 staining was localized to the cytoplasmic region of prostate cancer cells consistent with their known localization at the endoplasmic reticulum. Figure 1 shows examples of negative (H-score 0), low (H-score 1), intermediate (H-score 2) and high (H-score 3) expression of SOAT1 (Fig. 1 A–H) and SOAT2 (Fig. 1I–P). Both SOAT1 and SOAT2 were homogeneously distributed among the different cores in the TMA. Interrater reliability for assessing SOAT expression was strong and very strong with a κ-coefficient of 0.66 (95% CI 0.61–0.70) for SOAT1 and 0.91 (95% CI 0.89–0.94) for SOAT2, respectively.

Overall, 96% of SOAT1 and 95% of SOAT2 stained TMA cores could be assessed and SOAT1 and SOAT2 expression was detectable in more than 99% each. 25% of the samples showed strong (*n* = 73, H-score 3) and 75% weak (*n* = 219, H-score <3) SOAT1 expression. For SOAT2, 14% of the cores showed high (*n* = 40, H-score 3) and 86% low (*n* = 249, H-score <3) expression. SOAT1 and SOAT2 protein expression were moderately correlated (Spearman Rho = 0.339, *p* < 0.001).

### Impact of SOAT protein expression on BCR free survival

For survival analysis, only patients with complete clinical data and evaluable tissue were included. BCR was observed in 39 of 73 (53.4%) samples with high SOAT1 expression and 63 (28.7%) samples with low SOAT1 expression. 19 of 40 (47.5%) samples with high SOAT2 expression and 83 (33.3%) samples with low SOAT2 expression had BCR during a median follow-up of 89 months.

**Fig. 2:**
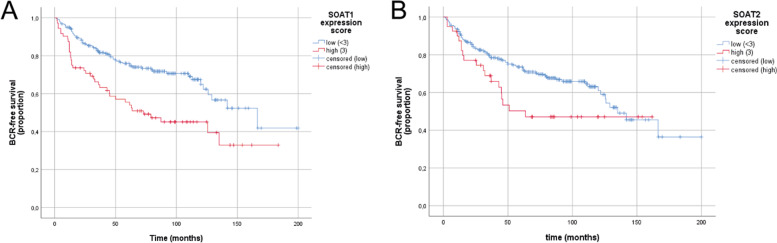
Kaplan-Meier plots of SOAT1 and SOAT2 on BCR. Strong SOAT1 expression (H-score 3, **A**) is highly associated with significantly shorter BCR free survival compared to low SOAT1 expression (H-score <3), Log-rank *p* < 0.001. SOAT2 expression (**B**) is not significantly associated with BCR-free survival. BCR-free survival = biochemical recurrence free survival.

Kaplan-Meier analysis revealed a significantly shorter median BCR free survival of 93 months (95% confidence interval [CI] 23.6-123.1 months) in patients with high SOAT1 (*n* = 72, Fig. 2A) compared to 134 (95% CI 112.6–220.2 months) in patients with low SOAT1 expression (*n* = 218, Log-rank *p* < 0.001).

Patients with high tumoral SOAT2 expression (H-score 3, *n* = 40, Fig. 2B) showed a trend for shorter BCR with a median of 91 months (95% CI not computed) compared to 125 (111.5–158.5 months) in patients with low SOAT2 staining (*n* = 247, Log rank *p* = 0.05).

To account for known prognostic factors in PCa including year of surgery, we performed Cox proportional hazards modeling with SOAT1 and age at surgery, pre-operative PSA, tumor stage, Gleason score, surgical margin status, lymph node status and year of surgery (Table 2). High SOAT1 expression retained significant association with less favorable BCR after multivariable adjustment (HR 2.40, 95% CI 1.57–3.68, *p* < 0.001). Earlier BCR in patients with Gleason score ≥8 approached statistical significance with a hazard ratio of 1.57 (95% CI 0.99-2.49, Log-rank *p* = 0.06). SOAT2 expression was not significantly associated with BCR (HR 1.57, 95% CI 0.93-2.64, Log-rank *p* = 0.09). SOAT staining and Gleason score were not significantly correlated (Pearson r = 0.042, *p* = 0.49).

**Table 2:** Impact of SOAT1 and SOAT2 expression on survival.

	Univariate analysis			Multivariate analysis SOAT1			Multivariate analysis SOAT2		
Variable	Mean survival (months)	95% CI	*p (Log-rank)*	HR	95% CI	*p (Cox regression)*	HR	95% CI	*p (Cox regression)*
**Age**									
<66.0	126	111–141	0.931						
≥66.0	112	100–124		1.10	0.60–1.37	0.64	0.85	0.56–1.29	0.43
**Pre-operative PSA** (Quartiles)									
≤21.38	104	87–121	0.054						
21.39–29.60	154	136–172		0.53	0.28–1.00	0.05	0.45	0.24–0.85	**0.01**
29.61–50.63	124	103–145		0.88	0.51–1.54	0.66	0.84	0.48–1.45	0.52
>50.63	101	83–119		1.24	0.73–2.10	0.43	1.04	0.62–1.76	0.88
**Clinical stage**									
T2 (58)	142	120–164	0.135						
T3(202)	116	103–128		1.57	0.88–2.87	0.13	1.32	075–2.34	0.34
T4 (43)	94	74–114		2.23	1.03–5.01	0.042	1.81	0.87–3.78	0.11
**Gleason score**									
≤7 (221)	131	118–144	0.115						
8–10 (63)	91	76–106		1.57	0.99–2.49	0.06	1.31	0.82–2.09	0.27
**Resection margins**									
R0 (121)	139	123–154	0.462						
R1 (147)	110	97–123		1.24	0.76–2.01	0.40	1.22	0.72–1.97	0.50
RX (35)	104	86–122		1.03	0.51–2.06	0.94	0.93	0.46–1.87	0.83
**Lymph node status**									
N0 (209)	121	107–133	0.190						
N1 (94)	126	109–143		0.80	0.50–1.31	0.39	0.70	0.43–1.17	0.17
**SOAT1 expression score**									
<3 (218)	134	120–148							
3 (72)	93	74–112	**<0.001**	2.40	1.57–3.68	**<0.001**			
**SOAT2 expression score**									
<3 (247)	125	113–139							
3 (40)	91	88–136	0.05				1.57	0.93–2.64	0.09
**Year of surgery**				1.00	0.93–1.08	0.967	0.97	0.90–1.05	0.44

Univariate survival analysis using Log-Rank Test and multivariate Cox-regression analysis showed a significant impact of strong SOAT1 expression score on biochemical recurrence free survival.

Statistically significant associations are highlighted in bold.

### Impact of SOAT1 and SOAT2 expression on secondary endpoints

Clinical recurrence-free survival was not significantly associated with high vs. low SOAT1 expression (median 167 months for both groups, Log-rank *p* = 0.816) nor was overall survival (high SOAT 1:172 months, low SOAT1: 174 months, Log-rank *P* = 0.476).

High vs. low SOAT2 expression was not significantly associated with clinical recurrence-free (162 vs. 171 months, Log-rank *p* = 0.346) and overall survival (177 vs. 171 months, Log-rank *p* = 0.962).

### Correlation of SOAT1 with lipid metabolism and androgen receptor (AR) signaling

In the TCGA and DreamTeam [20] cohorts, squalene epoxidase (SQLE) had a moderate significant positive correlation with SOAT1 (Spearman ρ = 0.20, *p* < 0.001 and *p* = 0.21, *p* < 0.01; Fig. S1A, B). In line with an increased lipid metabolism, low density lipoprotein receptor (LDLR) and SOAT1 were also moderately positively correlated (Spearman *p* = 0.38, *p* < 0.001 and ρ = 0.26, *p* < 0.001) in the TCGA and DreamTeam cohorts, respectively (Fig. S1C, D) and so were stearoyl-CoA desaturase (SCD) and SOAT1 (Spearman ρ = 0.25, *P* < 0.001 and ρ = 0.33, *p* < 0.001; Fig. S1E, F and Table S2). To explore the interconnection between SOAT1 expression and androgen receptor (AR) signaling on PCa progression [26] we examined the gene expression of SOAT1 and AR and found a good and statistically significant correlation in both the TCGA (Spearman ρ = 0.53, *p* < 0.001, Fig. S2A) and the DreamTeam cohort (Spearman ρ = 0.3, *p* < 0.01, Fig S2B). Accordingly, several AR signaling related genes [23] were consistently correlated with SOAT1 expression in both cohorts (Table S3).

## Discussion

PCa is the most frequent cancer in men but the overall mortality risk is low with only approximately 3% in western countries. However, about 15 to 30% of patients present with high risk PCa which is associated with distinctly higher aggressiveness and mortality rates. In addition, clinical outcome and likelihood of BCR vary due to different classification systems of high risk PCa [27–34]. Beyond established prognostic and clinical parameters such as Gleason score, PSA or tumor stage, reliable biomarkers for the prognostication of high risk PCa are still lacking, especially those further explaining high risk PCa biology. Yet, such prognostic and functional markers are crucial first to identify patients that are more likely to experience relapse and second to better stratify patients for personalized treatment approaches [19, 35]. Here, we evaluated the association of tissue protein expression of SOAT1 and SOAT2 in 305 high risk PCa patients on clinical parameters of recurrence.

While clinical progression and survival are more robust parameters of prognosis, biochemical recurrence is a surrogate marker of more aggressive clinical course and has been used in similar studies due to the small proportion of patients who experience one of those end points. In addition, biochemical recurrence impacts strongly on further treatment which may mask a biologically meaningful role of a given marker. Thus microRNA-221 has been identified as a prognostic marker and but is not ideally suited for routine clinical use.

Of note, high SOAT1 protein expression has already been demonstrated to correlate with a worse prognosis in hepatocellular carcinoma [36] and adrenocortical carcinoma [37].

Our data add high risk PCa to those malignancies where high SOAT1 expression is associated with earlier BCR. On the other hand, we did not observe association of SOAT1 expression with clinical recurrence and disease-specific death, most likely due to small percentages of patients who experienced clinical progression (14.4%) or died of high risk PCa (7.2%). Clinically, BCR triggers additional treatment that is likely to impact on clinical recurrence and survival.

SOAT1 catalyzes the esterification of free cholesterol to CE, which is subsequently stored in LDs. Free cholesterol accumulation is toxic e.g., through the induction of endoplasmic reticulum stress and hence cholesterol esterification favors cell survival. Abundancy of cholesterol has been shown to increase during PCa progression [38] and CE are enriched in LDs of human PCa tissues but not in normal prostates or benign prostatic hyperplasia [6]. Experimental inhibition of SOAT1 and consequently the esterification of free cholesterol reduced cell viability in a PCa cell culture model. Tumor growth [6] and metastasis was reduced in an orthotopic mouse model [7].

Our study now translates these experimental findings into a clinical setting and has the strength of a clinically well defined high risk PCa cohort which requires treatment and hence is not confounded by low-risk cases with a more benign course due to a different underlying tumoral biology. Although the two cohorts analyzed here were acquired and processed in two different centers and differ significantly in terms of variables associated with prognosis (Table S1), the high prognostic value of SOAT1 expression in the combined cohort strengthens its role of SOAT1 expression as factor of prognosis.

Data published by Stopsack et al. seemingly contradict our findings. Rather unexpectedly, they found low SOAT1 and low LDLR but high SQLE mRNA expression in clinical cohort studies to be associated with a higher Gleason score. Hence high mRNA expression of these genes was concluded to be associated with increased PCa dedifferentiation [39]. First, this study has the significant shortcoming that only mRNA expression was assessed which does not automatically correlate with protein expression and may largely be affected by stroma components [40, 41]_._ Second, decreased *SOAT1* gene methylation in PCa has been demonstrated which results in increased mRNA and protein expression in PCa [42]. Third, aberrant lipid metabolism is well established as a factor for PCa aggressiveness [43] and it would be surprising if low instead of high expression of the enzymes involved would be correlated with increased PCa dedifferentiation. The positive correlation of SOAT1 with enzymes involved in lipid metabolism (Fig. S1, Table S2) confirms our finding of high SOAT1 expression as a factor of poorer prognosis in high risk PCa as demonstrated for SQLE in another study [44]. In addition, our data suggest that SOAT1-dependent cholesterol metabolism and AR signaling may be functionally related and contribute to PCa progression. Importantly, increased intratumoral androgen synthesis from cholesterol associated with higher expression of genes involved in cholesterol metabolism have been shown [45] and potentially contribute to the development of castration refractory tumors.

The main limitation of our study is the retrospective study design and the limitation of samples to only two centers. Potential confounders such as statin use [46] but also follow-up treatment were unavailable which is expected to have varied over the relatively long recruitment period. Nevertheless, we consider the combined study cohort with its large sample number representative of the spectrum of high risk PCa. Importantly, very recently similar observations as ours have confirmed our findings [47].

While in the past, SOAT inhibition has been explored for the treatment of atherosclerosis [48], more recently, the SOAT1 inhibitor nevanimibe was tested in a phase I clinical trial as a treatment of adrenocortical carcinoma (ACC). Although the further development in ACC was discontinued due to the absence of clinically meaningful antitumoral activity, little dose-limiting-toxicities occurred and the drug was rather well tolerated [17]. Of note, SOAT1 expression was not reported in the study cohort although SOAT1 expression was shown to be very heterogenous in adrenocortical carcinoma [14, 37, 49]. Since in an exploratory experiment nevanimibe treatment of LNCaP (Fig. S3A) and PC3 PCa cells (Fig. S3B) significantly decreased viability, a clinical trial of the available SOAT1 inhibitor nevanimibe might present a possible treatment option in high risk PCa patients with high SOAT1 expression and biochemical progression.

## Conclusion

Our results clearly show a high prognostic value of SOAT1 protein expression in high risk PCa patients which helps to identify a subgroup of high risk PCa patients rather likely to experience BCR. SOAT1 expression may be associated with increased cholesterol metabolism and potentially androgen signaling in PCa. Patients with high SOAT1 expression might be good candidates for pharmacological SOAT1 inhibition. SOAT1 inhibitors have been clinically developed and may be repurposed for PCa treatment.

## Supplementary information

Supplemental Material

Supplemental Figure 1

Supplemental Figure 2

Supplemental figure 3

Supplemental Table 1

Supplemental Table 2

Supplemental Table 3
