# Supplementary material for: High expression of Sterol-O-Acyl transferase 1 (SOAT1), an enzyme involved in cholesterol metabolism, is associated with earlier biochemical recurrence in high risk prostate cancer
Source: Prostate Cancer Prostatic Dis. 2021 Jul 29;25(3):484–90. doi: 10.1038/s41391-021-00431-3 (PMC9385470; doi:10.1038/s41391-021-00431-3)
Supplement: Supplementary file 1 — Supplemental Material [file 41391_2021_431_MOESM1_ESM.docx]

Supplementary information

Unfavorable prognosis is associated with high Sterol-O-Acyl transferase 1 (SOAT1) protein expression in high risk prostate cancer

Carolin Eckhardt^1^, Iuliu Sbiera^1^, Markus Krebs^2,3^, Silviu Sbiera^1^, Martin Spahn^4,5^, Burkhard Kneitz^2^, Steven Joniau^6^, Martin Fassnacht^1,3,7^, Hubert Kübler^2,3^, Isabel Weigand^1,8^, Matthias Kroiss^1,3,7,8^

Material and methods

Cell culture

LNCap cells were cultivated in RPMI 1640 with 10 % FCS and PC3 cells were cultured in RPMI 1640 with 1 % NEA (non-essential amino acids), 1 mM sodiumpyruvate, 2 mM L-glutamine and 10 mM HEPES.

WST-1 Viability assay

Viability testing with WST-1 reagent was performed according to the manufacturer’s protocol (Roche) by employing a Victor_2_ multi-plate reader (Perkin-Elmer).

In silico analyses

mRNA expression data from the PRAD cohort (primary PCa) of the TCGA database and the DreamTeam cohort (PCa metastases) were accessed and correlation analyses were performed via cbioportal.org (Cerami et al. 2012, Gao et al. 2013)

Figure legends

**Supplementary figure 1:** Positive Pearson and Spearman rank correlation coefficients for SQLE and SOAT1 mRNA expression in PCa tissue in the PRAD cohort (primary PCa) within the TCGA database (A) and PCa metastases from the DreamTeam database (B). Positive Pearson and Spearman rank correlation coefficients for LDLR and SOAT1 mRNA expression in PCa tissue in the PRAD cohort (primary PCa) within the TCGA database (C) and PCa metastases from the DreamTeam database (D). Positive Pearson and Spearman rank correlation coefficients for SCD and SOAT1 mRNA expression in PCa tissue in the PRAD cohort (primary PCa) within the TCGA database (E) and PCa metastases from the DreamTeam database (F). SQLE: squalene epoxidase, LDLR: low density lipoprotein receptor, SCD: stearoyl-CoA desaturase

**Supplementary figure 2:** Positive Pearson and Spearman rank correlation coefficients for AR and SOAT1 mRNA expression in PCa tissue. (A) Data representing the PRAD cohort (primary PCa) within the TCGA database and (B) PCa metastases from the DreamTeam database.

**Supplementary figure 3:** Cell viability was reduced to approximately 50 % in LNCaP (A) and PC3 cells (B) after treatment with nevanimibe for 48h. ****:p<0.0001
