# Supplementary figures and images for: High expression of Sterol-O-Acyl transferase 1 (SOAT1), an enzyme involved in cholesterol metabolism, is associated with earlier biochemical recurrence in high risk prostate cancer
Source: Prostate Cancer Prostatic Dis. 2021 Jul 29;25(3):484–90. doi: 10.1038/s41391-021-00431-3 (PMC9385470; doi:10.1038/s41391-021-00431-3)

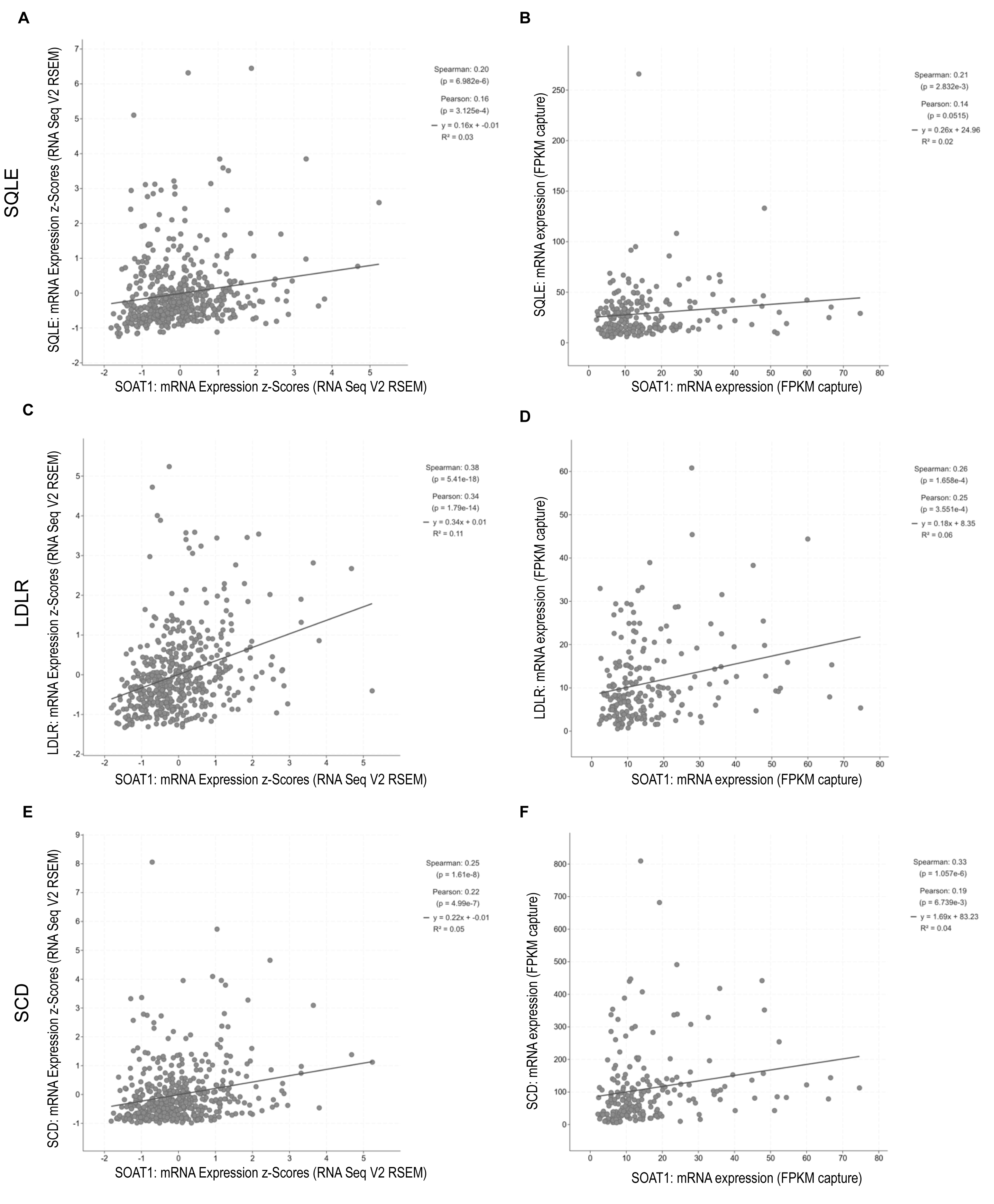

Supplement: Supplementary file 2 — Supplemental Figure 1 [file 41391_2021_431_MOESM2_ESM.tif]

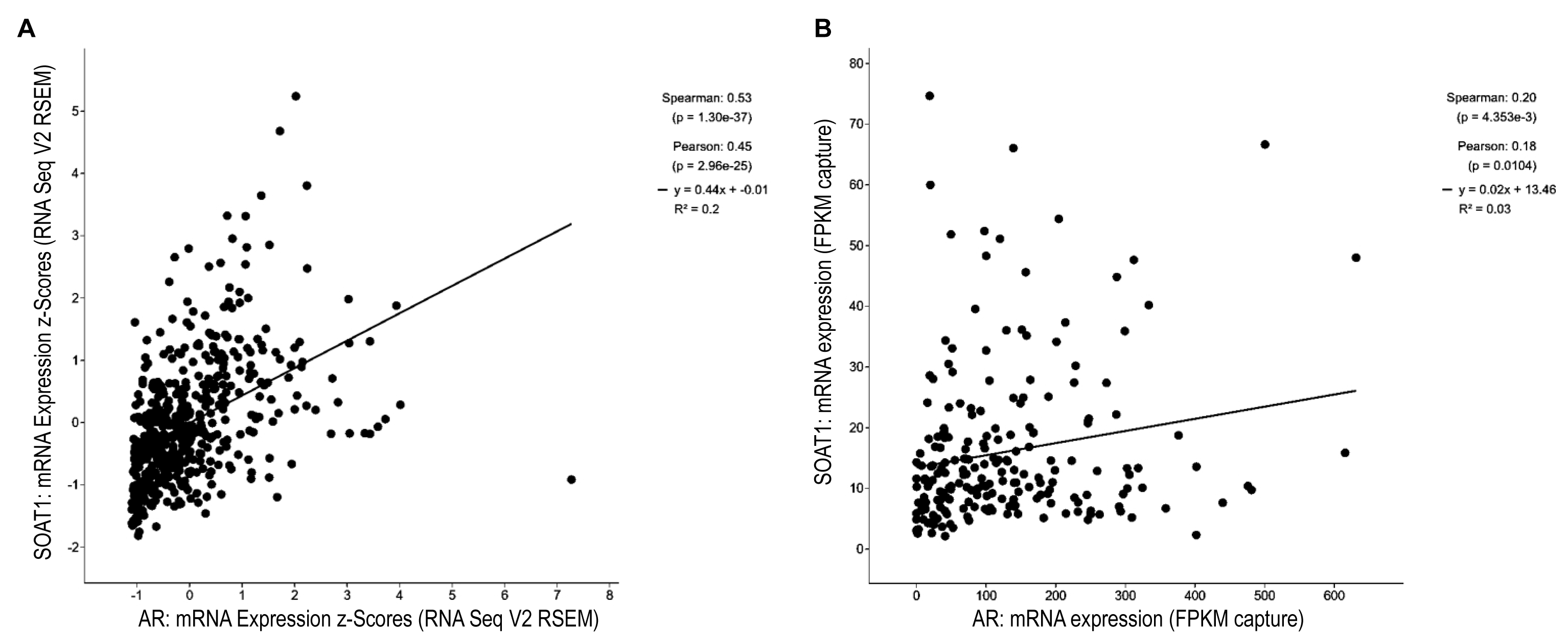

Supplement: Supplementary file 3 — Supplemental Figure 2 [file 41391_2021_431_MOESM3_ESM.tif]

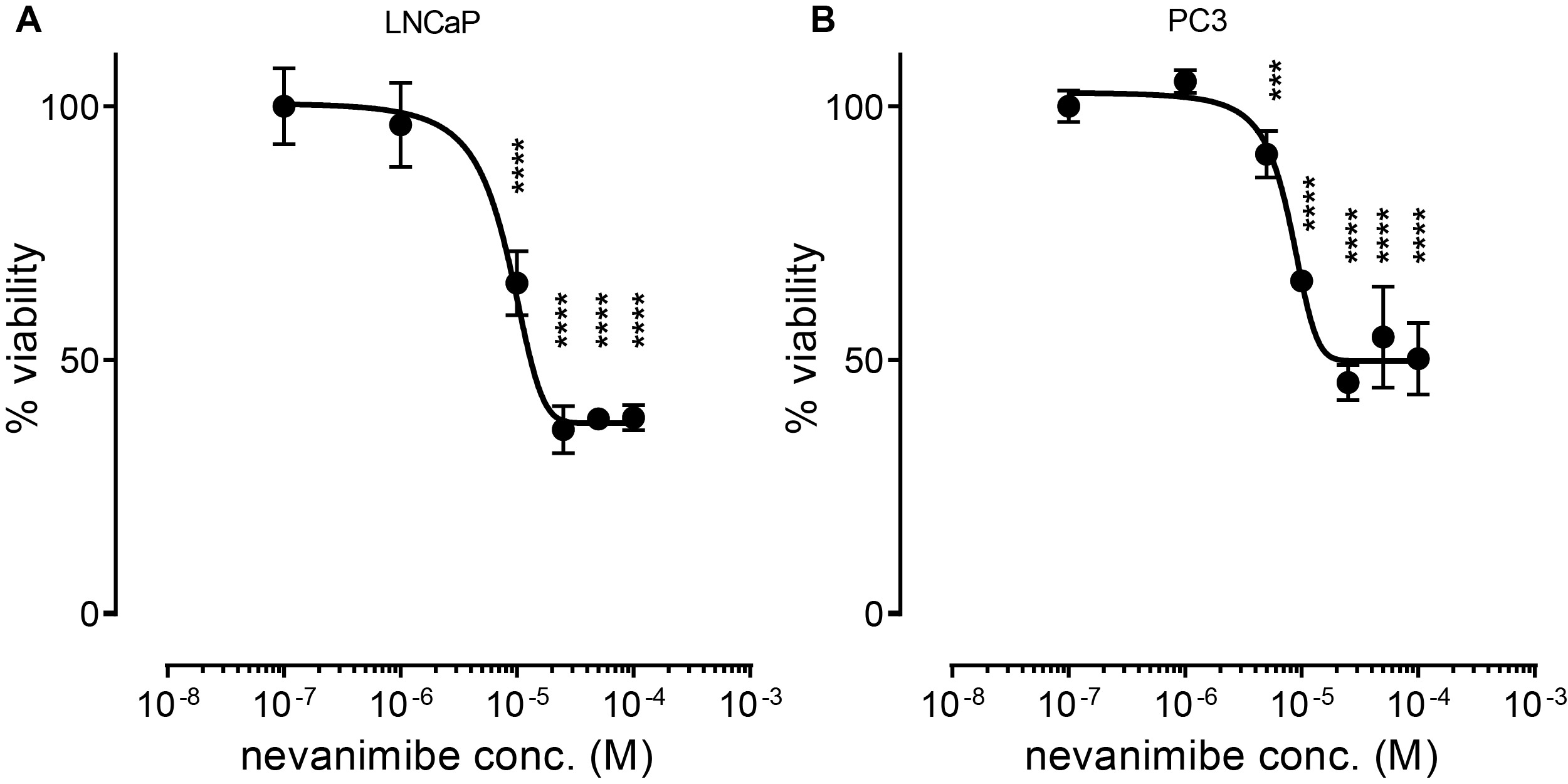

Supplement: Supplementary file 4 — Supplemental figure 3 [file 41391_2021_431_MOESM4_ESM.jpg]
