# Supplementary material for: High expression of Sterol-O-Acyl transferase 1 (SOAT1), an enzyme involved in cholesterol metabolism, is associated with earlier biochemical recurrence in high risk prostate cancer
Source: Prostate Cancer Prostatic Dis. 2021 Jul 29;25(3):484–90. doi: 10.1038/s41391-021-00431-3 (PMC9385470; doi:10.1038/s41391-021-00431-3)
Supplement: Supplementary file 5 — Supplemental Table 1 [file 41391_2021_431_MOESM5_ESM.docx]

| Variable | **Karlsruhe (A)** | *n* | *SD* | *mean* | **Leuven**  **(B)** | *n* | *SD* | *mean* |  |
| --- | --- | --- | --- | --- | --- | --- | --- | --- | --- |
| **Age** (range) | 67 (47-81) | *206* | *6.5* | *66.2* | 66 (41-76) | *99* | *8.2* | *64.0* | Mann-Whithney-U: p=0.08 |
| **Pre-operative PSA**, ng/ml (range) | 37 (20-597) | *206* | *49.0* | *50.9* | 20 (1-95) | *99* | *18.1* | *22.0* | Mann-Whithney-U: p<0.0001*** |
| **Clinical stage** | \|  \| \| --- \| |  |  |  |  |  |  |  |  |
| T2 | \| 33 (16.0%) \| \| --- \| |  |  |  | 26 (26.2%) |  |  |  |  |
| T3 | 136 (66.0%) |  |  |  | 67 (67.7%) |  |  |  |  |
| T4 | 37 (18.0%) |  |  |  | 6 (6.1%) |  |  |  | χ2=10.37  p=0.005** |
| **Gleason-Score** |  |  |  |  |  |  |  |  |  |
| ≤7 | 143 (76.5%) |  |  |  | 79 (79.8%) |  |  |  |  |
| 8-10 | 44 (23.5%) |  |  |  | 20 (20.2%) |  |  |  | χ2=0.41  p=0.52 |
| **Resection margin** |  |  |  |  |  |  |  |  |  |
| R0+RX | 106 (51.5%) |  |  |  | 52 (52.5%) |  |  |  |  |
| R1 | 100 (48.5%) |  |  |  | 47 (47.5%) |  |  |  | χ2=0.03  p=0.86 |
| **Lymph node status** |  |  |  |  |  |  |  |  |  |
| N0 | 124 (60.2%) |  |  |  | 87 (87.9%) |  |  |  |  |
| N1 | 82 (39.8%) |  |  |  | 11 (11.1%) |  |  |  | χ2=25.5  p<0.00001*** |
| **SOAT 1 expression score** |  |  |  |  |  |  |  |  |  |
| ≤3 | 167 (81.1%) |  |  |  | 52 (60.5%) |  |  |  |  |
| 3 | 39 (18.9%) |  |  |  | 34 (39.5%) |  |  |  | χ2=13.7  p=0.00021*** |
| **SOAT2 expression score** |  |  |  |  |  |  |  |  |  |
| ≤3 | 181 (87.9%) |  |  |  | 68 (81.9%) |  |  |  |  |
| 3 | 25 (12.1%) |  |  |  | 15 (18.1%) |  |  |  | χ2=1.74  p=0.18 |

**Supplementary Table 1: Comparison of clinopathological features between the two cohorts**

**Karlsruhe (A) and Leuven (B)**

BCR: biochemical recurrence
*= significant, **= very significant, ***= very very significant
