# Supplementary material for: High expression of Sterol-O-Acyl transferase 1 (SOAT1), an enzyme involved in cholesterol metabolism, is associated with earlier biochemical recurrence in high risk prostate cancer
Source: Prostate Cancer Prostatic Dis. 2021 Jul 29;25(3):484–90. doi: 10.1038/s41391-021-00431-3 (PMC9385470; doi:10.1038/s41391-021-00431-3)
Supplement: Supplementary file 6 — Supplemental Table 2 [file 41391_2021_431_MOESM6_ESM.docx]

**Table S3:** Spearman´s rank correlation coefficients for SOAT1 and androgen receptor (AR)-related genes (AR-related signature from Hieronymus et al. – DOI 10.1016/j.ccr.2006.09.005) within the prostate cancer cohort of the TCGA database and the Dream Team cohort. Significantly positive and negative correlation coefficients (after Benjamini-Hochberg correction) are highlighted in green and red, respectively. Data were accessed and calculated via cbioportal.org. National Center for Biotechnology Information (2021). PubChem summary for pathway R-HSA-191-273, Cholesterol Biosynthesis, Source Reactome. Retrieved April 09, 2021 from <https://pubchem.ncbi.nlm.nih.gov/pathway/reactome:R-HSA-191-273>

|  | **Spearman´s correlation coefficients - SOAT1 coexpression** | |  |
| --- | --- | --- | --- |
|  | PRAD cohort / TCGA n=491 | Dream Team cohort n=208 |  |
| PSA (KLK3) | 0.127 | 0.142 |  |
| TMPRSS2 | 0.228 | 0.228 |  |
| NKX3-1 | 0.268 | 0.32 |  |
| KLK2 | 0.046 | 0.055 |  |
| GNMT | -0.05 | 0.034 |  |
| TMEPAI (PMEPA1) | 0.23 | 0.311 |  |
| MPHOS9 (MPHOSPH9) | 0.41 | 0.136 |  |
| ZBTB10 | 0.451 | 0.221 |  |
| EAF2 | 0.137 | 0.065 |  |
| BM039 (CENPN) | 0.162 | 0.139 |  |
| SARG (C1ORF116) | 0.412 | 0.413 |  |
| ACSL3 | 0.512 | 0.243 |  |
| PTGER4 | 0.059 | 0.105 |  |
| ABCC4 | 0.419 | 0.178 |  |
| NNMT | -0.343 | -0.239 |  |
| ADAM7 | 0.066 | -0.215 |  |
| FKBP5 | 0.314 | 0.14 |  |
| ELL2 | 0.232 | 0.134 |  |
| MED28 | 0.169 | 0.005 |  |
| HERC3 | 0.384 | 0.204 |  |
| MAF | 0.049 | -0.059 |  |
| TNK1 | -0.063 | -0.031 |  |
| GLRA2 | 0.036 | -0.067 |  |
| MAPRE2 | 0.133 | -0.104 |  |
| PIP5K2B (PIP4K2B) | -0.064 | 0.187 |  |
| MAN1A1 | 0.264 | -0.115 |  |
| CD200 | -0.044 | 0.102 |  |
