# Supplementary material for: High expression of Sterol-O-Acyl transferase 1 (SOAT1), an enzyme involved in cholesterol metabolism, is associated with earlier biochemical recurrence in high risk prostate cancer
Source: Prostate Cancer Prostatic Dis. 2021 Jul 29;25(3):484–90. doi: 10.1038/s41391-021-00431-3 (PMC9385470; doi:10.1038/s41391-021-00431-3)
Supplement: Supplementary file 7 — Supplemental Table 3 [file 41391_2021_431_MOESM7_ESM.docx]

Table S2

Spearman´s rank correlation coefficients for SOAT1 and genes related to cholesterol biosynthesis (https://pubchem.ncbi.nlm.nih.gov/pathway/Reactome:R-HSA-191273) within the prostate cancer cohort of the TCGA database and the Dream Team cohort. Significantly positive and negative correlation coefficients (after Benjamini-Hochberg correction) are highlighted in green and red, respectively. Data were accessed and calculated via cbioportal.org.

|  | **Spearman´s correlation coefficients - SOAT1 coexpression** | |
| --- | --- | --- |
|  | PRAD cohort / TCGA n=491 | Dream Team cohort n=208 |
| ACAT2 | 0.12 | 0.014 |
| CYP51A1 | 0.301 | 0.28 |
| DHCR7 | 0.123 | 0.204 |
| DHCR24 | 0.327 | 0.17 |
| FDFT1 | 0.129 | 0.206 |
| HMGCS1 | 0.345 | 0.293 |
| LBR | 0.098 | 0.158 |
| LSS | -0.031 | 0.079 |
| MVD | -0.336 | 0.113 |
| MVK | -0.159 | 0.147 |
| MSMO1 | 0.239 | 0.211 |
| SC5D | 0.421 | 0.251 |
| SQLE | 0.201 | 0.206 |
| TM7SF2 | -0.248 | 0.255 |
| PMVK | -0.445 | 0.072 |
| EBP | -0.151 | 0.174 |
| NSDHL | -0.197 | 0.142 |
| HSD17B7 | -0.067 | -0.033 |
| ARV1 | 0.045 | 0.204 |
| PLPP6 | 0.35 | 0.225 |
|  |  |  |
|  |  |  |
